# Tumor Microenvironment

**DOI:** 10.3390/medicina56010015

**Published:** 2019-12-30

**Authors:** Borros Arneth

**Affiliations:** Institute of Laboratory Medicine and Pathobiochemistry, Molecular Diagnostics, University Hospital of the Universities of Giessen and Marburg UKGM, Justus Liebig University Giessen, Giessen, Germany, Feulgenstr. 12, 35392 Giessen, Germany; borros.arneth@klinchemie.med.uni-giessen.de; Tel.: +49-641-985-59248; Fax: +49-641-985-41579

**Keywords:** tumor-microenvironment, cancer-microenvironment, tumor-growth, tumor, cancer

## Abstract

*Background and Objectives:* The tumor microenvironment has been widely implicated in tumorigenesis because it harbors tumor cells that interact with surrounding cells through the circulatory and lymphatic systems to influence the development and progression of cancer. In addition, nonmalignant cells in the tumor microenvironment play critical roles in all the stages of carcinogenesis by stimulating and facilitating uncontrolled cell proliferation. *Aim:* This study aims to explore the concept of the tumor microenvironment by conducting a critical review of previous studies on the topic. *Materials and Methods:* This review relies on evidence presented in previous studies related to the topic. The articles included in this review were obtained from different medical and health databases. *Results and Discussion:* The tumor microenvironment has received significant attention in the cancer literature, with a particular focus on its role in tumor development and progression. Previous studies have identified various components of the tumor microenvironment that influence malignant behavior and progression. In addition to malignant cells, adipocytes, fibroblasts, tumor vasculature, lymphocytes, dendritic cells, and cancer-associated fibroblasts are present in the tumor microenvironment. Each of these cell types has unique immunological capabilities that determine whether the tumor will survive and affect neighboring cells. *Conclusion:* The tumor microenvironment harbors cancer stem cells and other molecules that contribute to tumor development and progression. Consequently, targeting and manipulating the cells and factors in the tumor microenvironment during cancer treatment can help control malignancies and achieve positive health outcomes.

## 1. Introduction

Cancer continues to place a significant economic and social burden on both developed and developing countries [1,2]. Approximately 14.1 million new cancer cases and 8.2 million cancer deaths were reported in 2012 [1]. The increasing prevalence and burden of known risk factors, such as physical inactivity, smoking, and obesity, will likely increase the number of new cancer cases locally and globally. In the long run, cancer contributes to increased mortality, poor health, and high healthcare costs [1,2]. Therefore, it is imperative to understand cancer development and progression to develop interventions that can be applied to promote the health and wellbeing of cancer patients [2,3]. According to Balkwill, Capasso, and Hagemann, tumors are masses of malignant cells that affect and corrupt the function and health of other cells in the body [4]. Both benign and malignant cells influence cancer progression and can lead to poor health and death [5,6]. Therefore, a review of the literature on the tumor microenvironment (TME) will improve the understanding of cancer [7,8]. In addition, it is imperative to explore the various interactions, processes, and cells in the TME. This review aims to examine the current knowledge of different aspects of the TME.

## 2. Materials and Methods

**Figure 1:**
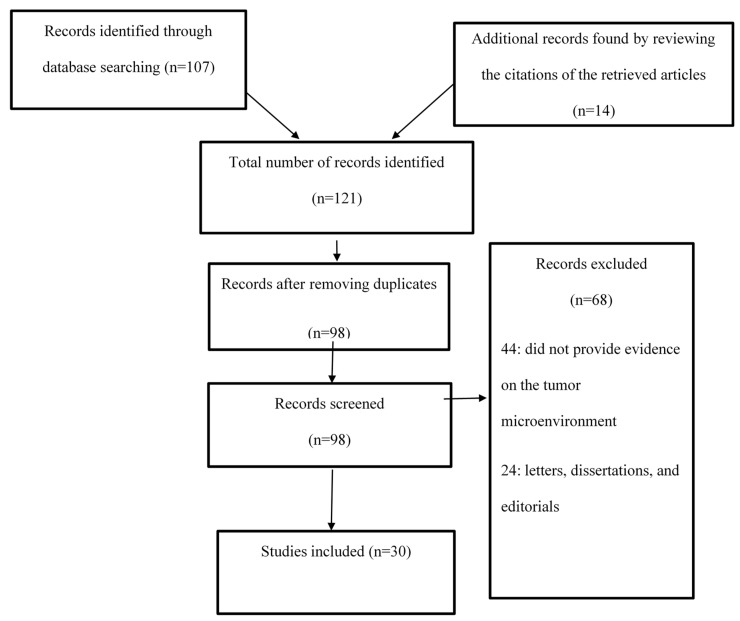
Prisma Flow Diagram.

The current study examined different aspects of the TME by reviewing the existing body of research evidence. The process entailed analyzing the results of studies on the TME in PsycINFO, CINAHL, PubMed, and Web of Science. Search terms and phrases such as “tumor microenvironment”, “cancer stem cells”, “cancer”, “TME and cancer”, “tumorigenesis”, “tumor cell growth”, “metastasis”, and “invasion” were used to identify articles that could help explore the research topic. The search was limited to articles that were published in English in the four aforementioned electronic databases between 2008 and 2017. The abstracts of the available articles were carefully reviewed to determine their quality and appropriateness, and the aim, research design, results, and conclusions in each of the selected articles were examined. Figure 1 shows a Prisma flow diagram for this study.

**Figure 2:**
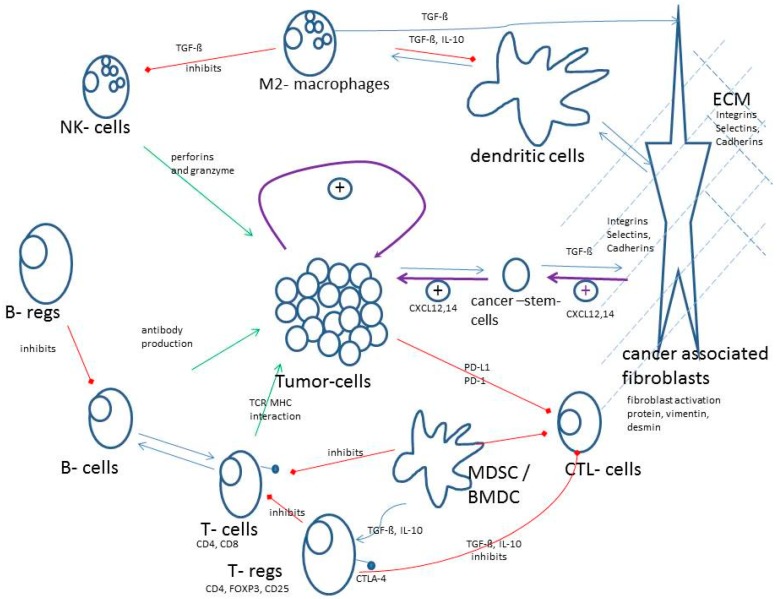
Schematic overview about the most important mechanisms and interactions of the tumor microenvironment (TME).

Figure 2 gives a schematic overview about the most important mechanisms and interactions of the TME. Green arrows indicate antitumor activities of the immune system and red arrows indicate inhibitions of antitumor activity of the immune system. Of course, tumors have strong positive tumor growing effects by themselves (loop in the middle).

## 3. Results

At the end of the search and review process, the final list of articles consisted of randomized controlled studies, meta-analyses, clinical trials, cohort studies, and systematic reviews. The articles were carefully examined for applicability to the study of the TME. A total of 30 articles were considered for review (Table 1).

## 4. Discussion

### 4.1. The Tumor Microenvironment

The TME refers to the cellular environment in which tumors or cancer stem cells exist. Cancer stem cells are cells in a tumor with the abilities to self-renew and drive tumorigenesis [8]. Previous studies have isolated unique cancer stem cells in samples from patients with breast, hematopoietic, colon, lung, and brain cancers [1,2,5]. These cells help improve the understanding of the TME [8,9], but pose significant challenges in the diagnosis and management of cancer. The TME encompasses the surrounding immune cells, blood vessels, extracellular matrix (ECM), fibroblasts, lymphocytes, bone marrow-derived inflammatory cells, and signaling molecules [9,10]. Interactions between malignant and nonmalignant cells create a TME that affects cancer development and progression [4,5]. The nonmalignant cells in the TME often play a protumorigenic function at all phases of carcinogenesis by stimulating uncontrolled cell proliferation [6,7,8]. In contrast, malignant cells invade healthy tissues and spread to other body parts through the lymphatic or circulatory system.

The TME comprises different cellular components. The first is endothelial cells, which play a key role in tumor development and tumor cell protection from the immune system. Tumor angiogenic vessels usually branch outwards from preexisting vessels or are derived from endothelial progenitor cells [6]. In this way, these cells offer nutritional support for tumor growth and development. The second major component is immune cells, such as granulocytes, lymphocytes, and macrophages. These cells are involved in various immune responses and activities, such as inflammatory reactions orchestrated by the tumor to promote survival. The most prominent immune cell type in the TME is the macrophage [6,7]. Macrophages have diverse functions that are linked to cancer development and progression; they promote the escape of tumor cells into the circulatory system and can suppress antitumor immune mechanisms and responses [7]. Evidence from previous studies has revealed that macrophages can help circulating cancer cells extravasate at distant sites, such as the lungs, which can lead to the persistent growth of metastatic colonies [8,9,10]. An increasing number of studies have revealed that tumor-associated macrophages (TAMs) can augment, mediate, or antagonize the antitumor activity of irradiation, cytotoxic agents, and checkpoint inhibitors. The final cell type in the TME is the fibroblast [11,12,37]. Fibroblasts allow cancer cells to migrate from the primary tumor location into the bloodstream for systemic metastasis. Furthermore, fibroblasts provide a reliable passage for endothelial cells undergoing angiogenesis in the tumor.

The role of the ECM in cancer development and progression has been examined in previous studies. The ECM consists of a network of macromolecules, including glycoproteins, collagens, and enzymes, that support biomechanical activities and functions in the body [6,8]. Importantly, the ECM is composed of active tissue components that influence cell adhesion, proliferation, and communication [2,12,13]. The cellular growth factors found in the matrix near other cell membranes, such as integrins, are implicated in the ability of cells to communicate with the TME. The ECM further influences the migration of cancer cells by altering its physical properties, composition, and topography [14]. The adhesion gradient and the ECM concentration determine the speed at which cancer cells migrate from one region to another.

Some recent empirical studies and reviews have shown that the TME harbors carcinoma cells and sarcoma cells, as well as hematopoietic, mesenchymal, and noncellular components, that contribute to tumor heterogeneity [12,37]. For example, LeBleu noted that the TME represents a complex and dynamic milieu of both cellular and acellular components with synergistic responses and functions in cancer progression [11]. According to Korneev et al., tumors usually interact closely and continuously with the surrounding microenvironment and organs via the lymphatic or circulatory system [12]. Furthermore, tumor cells can influence the microenvironment through the release of extracellular paracrine signals that induce peripheral immune tolerance and support tumor angiogenesis [12]. Similarly, immune cells in the TME can affect the evolution, growth, and progression of cancerous cells in patients suffering from different types of cancer [11].

In other cases, the TME is regarded as a heterogeneous, complex, and dominant component of a solid tumor [11]. Therefore, understanding the processes and interactions that occur in the TME is critical for the management of cancer and the interpretation and analysis of imaging data [11,13]. For instance, understanding the role of the naïve stroma will help caregivers determine how the TME influences the physiological homeostasis of normal tissues [12,14,15]. A study by Rupp et al. found that the stroma affects physiological homeostasis by controlling tumorigenesis, tumor cell growth, metastasis, and invasion [14]. Moreover, the stroma contributes to the anchorage-independent progression and growth of mesenchymal cells [14]. These findings support the long-held notion that the heterogeneous and complex components of the TME contribute to the development of cancer.

The TME has also been studied with regard to how cell infiltration and angiogenesis affect the progression of organ- and tissue-specific tumors [15,16,17]. According to Pujari and Vidya, angiogenesis and the presence of immune cells in the TME lead to the generation of cancer stem cells while providing a complex signaling cellular environment [16]. Furthermore, cancer stem cells act as tumor-initiating cells that support the spread of cancerous cells and lead to therapeutic resistance [16] through various intrinsic mechanisms, including epigenetic and genetic changes [16,17,38]. In addition, cancer stem cells have been implicated in TME-mediated signaling processes, such as immune modulation and the formation of inflammatory niches [18,38,39].

There is consensus among researchers that the composition and structure of the TME vary among cancer types and among patients. In addition to multiple malignant cells, the TME harbors secreted proteins, blood vessels, and nonmalignant cells that support and influence tumor growth. Bussard et al. stated that the TME contains a heterogeneous population of cancer cells and nearby endogenous stromal cells that have been recruited to facilitate tumor progression [18]. However, it is becoming well established that the actual composition of the microenvironment, as well as how it shapes cancer development and progression, can vary among patients and types of cancer [18].

### 4.2. Immune Tumor Microenvironment

Recent studies have shown that the recruitment, successful activation, and reprogramming of immune and stromal cells in the extracellular space are outcomes of reciprocal interactions between cancer cells and the TME [19,20]. Furthermore, it has been reported that cancer development and progression are influenced by components of the TME and controlled by the host immune system [20]. Therefore, TME components and immune system biomarkers are important for cancer detection and evaluations of prognoses and treatment response [19,20,21]. The examination of the immune TME has critical prognostic value, and can supplement histopathological and molecular biomarkers with regard to the evaluation of patient responses to treatment.

For cancer cells to progress into a potentially lethal and clinically-relevant disease, they must gain the ability to escape epithelial compartments and systematically invade other cellular spaces [20]. This escape requires that tumor cells degrade the basement membrane and separate the tissue parenchyma from the epithelial compartment [20]. Additionally, during the invasion process, tumor growth is partially influenced and controlled by the TME through paracrine and juxtacrine interactions [20]. In such cases, the tumor-associated stroma provides nutrients, oxygen, enzymes, and matrix-bound growth factors that facilitate tumor progression and proliferation [20].

Research has shown that cellular interactions in the TME affect cancer development and progression. During the initial stages of tumor development, malignant cells in the TME are poor stimulators and poor targets of the immune response. With time, these cells become resistant to the innate immune response and then begin to impair the adaptive immune response [21,40,41]. The process of undermining these immune responses entails blocking the function and maturation of T lymphocytes, which eventually form a significant part of the TME. However, previous studies tend to contradict each other regarding the exact effects of specific T cells. Some T cells are reported to promote tumorigenesis, whereas others are tumor restrictive [40,41,42]. One common type of T lymphocytes in the TME is cytotoxic CD8^+^ memory T cells, which are capable of contributing to excellent cancer prognoses by killing tumor cells [42]. Cytotoxic CD8^+^ memory T cells kill tumor cells by recognizing a specific antigen on the tumor cells and stimulating an immune response, which follows a prototypical, tri-phasic pathway [43]. CD8^+^ T cells in the TME are usually supported by CD4^+^ T helper 1 (TH1) cells that release interferon-gamma (IFN-γ) and interleukin-2 (IL-2) [41]. Other CD4^+^ cell populations, such as TH2 cells, support the B cell response through the production of IL-4, IL-5, and IL-13 [21,22]. TH17 cells, on the other hand, produce IL-17A, IL-17F, IL-21, and IL-22, which promote tumor growth by favoring antimicrobial tissue inflammation [22,43].

The importance of the TME in tumor development has also been discussed based on the role of B lymphocytes in innate natural killer T (NKT) and natural killer (NK) cells. B lymphocytes are commonly present in draining lymph nodes and lymphoid structures adjacent to the TME and the invasive tumor margin [4,23]. The B lymphocytes in the TME play critical roles in both the regulation of tumor cell survival and proliferation and the development of treatment resistance. In other cases, these cells have been linked to the process of fostering immune escape [4]. The actual role and impact of these cells in relation to cancer development and tumor suppression remain major topics for research and discussion due to different underlying mechanisms [24,44]. However, controlling B cells in the TME helps interrupt the initiation of cancer-induced immunosuppressive events, such as the TGF-β-dependent conversion of FoxP3^+^ cells to support and promote metastasis [45,46]. NK and NKT cells, on the other hand, express and use inhibitory, adhesion, activating, and cytokine receptors to identify cellular targets and spare healthy cells [25]. The signals from these receptors can be transduced simultaneously during contact with cells in the TME to dictate the activation of NK cells [25]. Other recent studies have shown that NK and NKT cells detect internal changes in host tissues through “stress-induced” self-recognition [25,47,48]; these cells can also lead to the downstream activation of adaptive and innate immune cells in the TME [48].

TAMs, dendritic cells, and cancer-associated fibroblasts (CAFs) are the other cells that determine the significance of the TME in tumor development and progression. Some studies have reported that TAMs support cell invasion and expansion through the production of various molecules that promote tissue remodeling, such as EGF, MMP9, MT1-MMP, and MMP2, and of proinflammatory molecules, such as TNF-α, CXCL10, and IL-1β [48]. Additionally, these cells have immune functions and can release growth factors and cytokines in response to cancer cells, thus facilitating tumor cell proliferation, migration, and survival [49]. Other studies have argued that TAMs usually express the adhesion molecule Vcam1 and can proliferate upon differentiation into inflammatory monocytes [50]. Dendritic cells in the TME contribute to antigen presentation and processing by acting as messengers between the adaptive and innate immune systems [51,52]. These cells also migrate to lymph nodes, where they shape and initiate an adaptive immune response by stimulating T cells and B cells [26]. Interestingly, CAFs contribute to tumor cell proliferation by maintaining continuous propagation and growth at metastatic sites [53,54]. In addition, these cells secrete cytokines and growth factors, such as fibroblast secreted protein-1 (FSP1), that initiate metastatic colon and breast cancer [54,55,56].

In patients suffering from cancer, regulatory T cells (Tregs) can suppress antitumor immune responses, thus facilitating the development of an immunosuppressive TME and promoting cancer progression [57]. These cells have been characterized extensively in both immune infiltrates and peripheral blood samples from patients with different types of cancer. Recent research has shown that FoxP3^+^ Treg accumulation within the TME can indicate a worse prognosis of cancer patients, including those suffering from ovarian and pancreatic ductal adenocarcinomas [57]. Clinical studies have shown that Treg depletion can induce the regression of metastatic lesions in patients with advanced-stage melanoma [57]. Furthermore, Treg depletion and subsequent cancer antigen vaccination can initiate antitumor CD4^+^ T cell responses.

### 4.3. Does the Tumor Influence its Environment?

The TME is a dynamic and complex milieu of various stromal cells that surround cancer cells. The cells within the TME interact with each other and with cancer cells to influence tumor development and progression [27]. In the process, they influence cancer cell invasion, tumor growth, and metastasis. Recent studies have reported that the TME remains an essential battlefield between the host immune system and the tumor [27,57]. The broad range of cellular interactions in this particular environment will determine the host’s tolerance and response to the tumor. Recent advances in cellular tumor and molecular immunology have revealed that tumors affect and influence the TME through the active recruitment and modulation of various cell phenotypes and functions [28,58], with the goal of promoting immune suppression and tolerance to tumor-associated antigens [28,29].

Mesenchymal cells are crucial players in the interactions between tumors and the TME [30,59,60]. This particular type pf stromal cell can influence various aspects of tumor biology due to its ability to differentiate into pericytes and CAFs [30,60]. The functions of these heterogeneous groups of cells can be pirated by cancer cells and redirected towards carcinogenesis [53]. Attempts have been made to identify the different chemotactic factors released by tumor cells to facilitate the recruitment and corruption of mesenchymal cells [31,60]; some of the commonly identified factors are peptide signaling molecules, stromal cell-derived factor 1 (SDF-1), monocyte chemoattractant protein 1 (MCP-1), leucine-37 (LL-37), and transforming growth factor β (TGFβ) [31,60]. In other cases, signaling molecules such as nitric oxide (NO) and exosomes have been identified as tumor-secreted factors that influence the TME [31,60].

Studies have also shown that tumor cells can activate fibroblasts, thus promoting the development of cancer. However, the pathway underlying the stromal activation of fibroblasts in the TME is not fully understood [59,60]. Evidence from animal model studies suggests that it may involve prostaglandin (PG) E2 activation and Wnt signaling [30,31]. Furthermore, researchers hypothesized that fibroblasts may be activated by vascular endothelial growth factor A (VEGFA) signaling, thus inducing cancer development [31,61]. Therefore, stromal activated fibroblasts play an important role in tumor growth, and can be targeted for the treatment of different types of cancer.

### 4.4. Stem Cells and Their Protection by the Tumor Microenvironment

The TME is usually composed of a wide range of cancer cells that facilitate tumor heterogeneity. Cancer stem cells usually interact with the TME through the activation of self-renewal and stem cell-related pathways, such as the Notch-1 and PI3K pathways [29,59]. Furthermore, cancer stem cells can survive under hypoxic conditions by promoting the production of hypoxia-inducible factor (HIF)-1α, VEGF, and proangiogenic factors. In some cases, stem cells induce immune tolerance within the TME through the production of anti-inflammatory cytokines [60]. Therefore, targeting such pathways could help manage different types of cancer and reduce the likelihood of disease recurrence.

In some cases, the protection of cancer stem cells by the TME involves CAFs and epithelial-to-mesenchymal transition (EMT) [30,31,61]. CAFs are an active stromal component of the TME that can drive tumor progression through the secretion of soluble factors, modulate the composition of the ECM, and interact with other cell types. Furthermore, these cells are capable of orchestrating tumor-like behavior in the TME through the secretion of exosomes that eventually stimulate cell migration [62,63]. In patients who have prostate cancer, CAFs in the TME can promote the growth of cancer stem cells by increasing cell proliferation and spheroid formation via paracrine signaling. EMT refers to the process through which epithelial cells become fibroblast-mesenchymal cells [61,62]. In the TME, increased cell invasiveness and motility and the turnover of ECM components accompany EMT. The TME protects cancer stem cells via EMT by enabling them to invade basement membranes, move to distant sites, and form secondary tumors.

Researchers have identified and studied cancer stem cells in samples collected from people with multiple solid and hematological cancers, including those of the breast, prostate, head and neck, and ovaries [61,62]. The results of these studies reveal that cancer stem cells are a general feature of many types of cancer. The analysis of cancer stem cells in the TME has provided new strategies for examining cancer treatments, stromal components, and invasion processes [61]. Furthermore, researchers have found that the TME uses a wide range of mechanisms to protect cancer stem cells [61]. The findings show that cancer stem cells are an attractive target for the development of new cancer therapies.

Tumor cells are usually embedded in a dense ECM composed of collagen, proteoglycans, proteins, and glycoproteins [62]. The increased matrix deposition of these substances may hinder the efficacy of anticancer agents such as biologics and chemotherapy. Furthermore, the presence of collagen and hyaluronan in the TME increases tension in the ECM [61]; these substances increase growth-induced solid stress and put significant pressure on blood cells. Previous studies have revealed that the TME may have a direct effect on cancer stem cells via molecules such as MMP-3 and Wnt ligands [61,62].

In some cases, the TME can protect cancer stem cells through angiogenesis and metabolism-related mechanisms [61,62]. Tumoral angiogenesis requires an association between endothelial cells and pericytes, and occurs in a disorganized and rapid manner that can lead to convoluted blood flow. Rapid cellular proliferation, coupled with a high oxygen consumption rate, may lead to inadequate nutrient delivery to the tumor, which can cause hypoxia and compressed blood vessels. HIF1α is activated by hypoxia and regulates alternative angiogenic signaling processes [61,62]. The insufficient blood flow in the TME as a result of disorganized angiogenesis can create a demanding yet unique metabolic environment. Additionally, the concentration of energy sources in the tumor region can force cancer cells to rely on glycolysis for energy to proliferate and execute effector functions, such as the release of IFN-γ [63]. Recent investigations have shown that T cells and glycolytic cancer cells in the TME can upregulate the expression of various glucose transporters, such as SGLT-1 and GLUT-1 [61,64]. In some cases, activated T cells can take up glucose within the tumor environment without significant competition from tumor cells.

Mesenchymal cells and fibroblasts may also be involved in the protection of cancer stem cells by the TME. Stromal cells are known for their ability to divide and differentiate into various lineages depending on the composition of the TME [61]. Additionally, the TME contains many growth factors, such as granulocyte-macrophage colony-stimulating factor (GM-CSF) and VEGF, that can reduce antitumor T cell activity in metastatic melanoma [61,62]. This finding indicates that the mesenchymal components of the TME are involved in supporting human tumor progression. Recent investigations have shown that leukocytes and factors such as TGF-β, GM-CSF, and PDGF tend to promote tumor growth, increase angiogenesis, and hinder the release of signaling molecules [61]. In this regard, it is apparent that the TME offers an important shelter for cancer stem cells by regulating signaling molecules and immune cell activity [61,62]. Hence, there is a need to conduct further comprehensive investigations on how to outpace tumor evolution and to develop both biologic and pharmacologic interventions that can help eradicate cancer stem cells.

Controlling the proliferation of cancer stem cells is not easy, and this challenge is made more difficult by the protective effect of the TME, which consists of infiltrating endothelial cells, immune cells, signaling molecules, and ECM [65]. The TME tends to act as a therapeutic barrier that promotes tumor development and progression due to its immunosuppressive capabilities [64]. Researchers have reported that the recruitment of cancer stem cells to the tumor site occurs because of the favorable, hypoxic conditions in the TME [63,64]. In addition, TME components usually secrete cytokines, growth factors, and chemokines that promote tumor cell migration [65,66,67]. In this sense, the TME protects cancer stem cells from both host anticancer immunity and pharmaceutical interventions applied to control cancer growth [32,68]. Table 2 provides a summary of some of the primary cells and molecules in the TME.

### 4.5. The Role and Functions of the Extracellular Matrix (ECM) and Integrins

The ECM is considered the noncellular component of tissue that offers structural and biochemical support for cellular components. Instead of acting merely as an intercellular bulk, the ECM has physiological capabilities similar to those of a living cell [14], allowing it to influence cell communication, adhesion, and proliferation. A review of studies shows that the ECM is composed of water, fibrous proteins, proteoglycans, and minerals [14]. The unique composition of the matrix is attributed to biochemical and biophysical feedback that takes place between cellular components and the microenvironment in which tissues develop [14,20]. The components of the ECM may differ, depending on the resident cells and the needs of the specific tissue. Recent studies have shown that the ECM regulates the production of different fibrous proteins, including laminin, elastin, and collagen [12,59]. Although the ECM is highly dynamic, it can undergo a remodeling process in which its primary components are modified and degraded, aided by ECM proteinases. The ECM may function as a track for cell migration and proliferation, which is influenced by topography, physical properties, and cell composition. Research evidence shows that cells can migrate from regions with a low ECM concentration to those with a high concentration as a result of the adhesion gradient [12]. In the context of cancer development, however, the relationship may be nuanced, as ECM concentrations that are too high may hinder cell migration.

Recent studies have further revealed that the ECM acts as a TGFβ reservoir [12,59]. TGFβ can regulate the expansion of neural and epithelial cells, wound repair, and immune responses. In some cases, however, these regulatory processes are adversely affected by events that cause signaling pathway malfunctions, such as tumorigenesis. Almost all human cells are responsive to TGFβ due to its critical role in maintaining tissue homeostasis and preventing the progression of incipient tumors [59]. However, cancer cells are genetically unstable and can evade the suppressive effect of the TGFβ pathway in the TME. In particular, malignant cells tend to circumvent the TGFβ pathway by inactivating its core components, such as TGFβ receptors, or by disabling the tumor-suppressive components and arms of the pathway. Other studies have shown that TGFβ can enforce immune tolerance [12]. Therefore, tumors that produce TGFβ may be shielded from immune surveillance. In addition, tumor-associated TGFβ may help recruit stromal cells, such as myofibroblasts and osteoclasts, thus promoting tumorigenesis.

Finally, the composition of the ECM and its biomechanical characteristics affect integrin signaling, thus influencing important processes that affect cancer development, such as the Hippo pathway and EMT [12]. Cells usually attach to the ECM via a wide range of receptors, including integrins, which play a critical role in promoting epithelial differentiation and cell development [68,70]. Additionally, the loss of integrin subunits, such as α6 and α2, can promote tumor progression. Integrin function and activity are dependent on substances such as syndecans, which bind ECM proteins such as collagen and laminin. These processes can compromise signaling pathways such as EMT during cancer development.

### 4.6. Drugs and the Tumor Microenvironment

The TME has become an area of intense interest due to its possible role in tumorigenesis and the fact that it provides an avenue through which to control tumor cells [70]. Furthermore, the TME has become a focal point of research, with attempts to develop drugs and interventions to manage and treat cancer [33,71]. Currently, surgery, chemotherapy, and radiotherapy remain the most common treatment options for solid tumors. However, a comprehensive understanding of the TME has contributed to the development of novel therapeutics and strategies for managing cancer. For instance, the selective depletion of Tregs in the TME can augment the function and generation of vaccine-elicited CD8^+^ memory T cells in patients with cancer [34,69]. However, studies have found that Tregs can improve prognosis by suppressing the growth of tumor cells [34,69]. More specifically, in Hodgkin’s lymphoma, Tregs improve patient survival through the direct suppression of tumor cell division and growth [35,72]. In any case, Tregs are an excellent target for anticancer therapeutics. Research attention has also focused on controlling the protumorigenic activity of cells in the TME, such as NK and NKT cells [36,72,73]. Additionally, the functions of mesenchymal cells, such as increasing the number of tumor-initiating cells and VEGF expression, have emerged as essential targets for the development of cancer drugs [74]. Drugs that regulate these functions can be used to promote tumor immunosurveillance and to treat different types of cancer.

### 4.7. Tumor Acidosis

Cancer is largely considered a disease of gene mutations and alterations. However, researchers have also shown that cancer is defined by massive and significant metabolic programming that adversely affects normal body functions [75]. This reprogramming is often complex, and can involve metabolic cooperativity between the surrounding stroma and cancer cells [76]. One area in which researchers have focused regarding metabolic adaptations in cancer is the acidification of the TME. There is consensus among researchers that acidosis is critical for malignant progression and somatic evolution [75]. Furthermore, acidosis can influence malignant behaviors, determine the metastasis and invasion rates, and dictate the mechanism of immunosurveillance. In this regard, tumor acidosis appears to be an important therapeutic target for the management of cancer.

Tumor acidosis has been recognized as a major hallmark of cancer development that can influence the treatment response and the severity of symptoms [76,77]. Furthermore, acidosis is no longer perceived as a passive collateral effect of tumor growth; rather, it is considered an important regulator of tumor progression. A review of research evidence shows that tumor acidosis can be linked to extracellular lactic acid accumulation and hypoxia [75]. The high metabolic demands of tumor cells often lead to the significant accumulation of H+ within the TME. Additionally, the disorganized nature of the tumor vasculature usually prevents the effective and timely elimination of H+ ions from the extracellular medium [76], leading to the development of hypoxic regions in the TME and a shift in glycolytic metabolism. In other instances, the buildup of H+ ions is associated with the hydration of carbon dioxide in oxidative tumor regions. These events usually occur at a high rate to fulfill the biosynthetic and bioenergetic needs of tumors.

In the last decade, there have been numerous publications associating tumor acidosis with various features of cancer development and progression, such as distant metastatic spread and local tumor invasion. Recently, researchers have reported that decreasing the pH in the TME can increase cancer cell motility and cause changes in cytoskeletal dynamics that affect the polarization and activity of macrophages and fibroblasts [75]. The alkalization of the intratumoral pH may contribute to increased cell migration through the involvement of actin-binding proteins [76]. Extracellular acidification, on the other hand, can result in protease activation and cell-cell interactions. Tumor areas with the lowest pH have been found to be associated with the highest rate of tumor invasion, and vice versa.

The relationship among lysosomal proteins, autophagy, and TME acidification has attracted the attention of researchers. Available research suggests that lysosome-associated membrane protein 2 (LAMP2), for instance, is a crucial protein that contributes to cancer cell survival under acidosis [75,76]. LAMP2 can protect lysosomal membranes from acidic proteolysis during cancer development. Increased acidity in the TME can lead to the expression of the autophagy regulator autophagy-related 5 (ATG5) in preinvasive cancer cells. In addition, cells chronically exposed to low pH have increased levels of autophagy biomarkers such as ATG5 and BCL-2 [75]. However, the actual mechanism through which these changes occur is not yet fully understood. Nevertheless, tumor acidosis is regarded as a critical therapeutic target for the development of new interventions. Targeting this entity may involve considering the metabolic vulnerabilities related to acidosis, neutralizing the acid with buffers, inhibiting the production of hydrogen ions, and understanding the possible role of nanomedicines in cancer management.

### 4.8. Immune Checkpoint Inhibition

The other area of research focus with regard to the TME and cancer management is immune checkpoint inhibition. Research has shown that immune checkpoint inhibitors, such as programmed death ligand 1 (PD-L1) on tumor cells and programmed death 1 (PD-1) on normal cells, help maintain immune responses [13]. Tumor cells express PD-Ligand (PD-L1), which binds onto and activates PD-1. The binding of PD-L1 to PD-1 inhibits the immune reaction of the cell on which PD-1 is expressed.

Checkpoint inhibitors are considered an emerging and important approach to the frontline management of various cancer types. PD-1 and PD-L1 inhibitors can also hinder the association of PD-L1 with its cellular receptors [13]. These inhibitors have been tested clinically in advanced-stage melanoma, renal cell carcinoma, non-small cell lung cancer, colon cancer, and bladder cancer [13,76,77]. Immunotherapies based on checkpoint inhibitors tend to shrink tumors in patients with different cancers, and are linked to durable responses and low toxicity levels. Therefore, they are regarded as promising interventions that can help manage cancer.

### 4.9. Targeting ECM-Integrin Signaling

Researchers have further explored the possibility of developing drugs that target ECM-integrin signaling to manage cancer [78,79]. This interest is based on studies showing that integrins are primary cell surface receptors for different ECM proteins that can influence and mediate a broad spectrum of cellular functions, such as differentiation, proliferation, and survival [79]. Today, integrins and their signaling effectors are among the most promising markers and targets in cancer management. Additionally, significant progress has been made with regard to developing mechanisms for targeting integrins in patients with cancer [79]. One example is the use of integrin antagonists that successfully block tumor progression. Other interventions, such as the avβ3 and avβ5 integrin antagonist cilengitide, have shown important anticancer clinical activity. Further investigations are needed to explore the efficacy of these interventions and their potential side effects in the treatment of specific types of cancer.

### 4.10. Transforming Growth Factor-Beta (TGF-Beta) and Activin Targeting

Cancer is not just a condition characterized by a mass of malignant cells; it is a disease associated with complex rogue tissues and organs corrupted by malignant and transformed cells in the TME [76]. Additionally, disease progression occurs as a result of dynamic and complex communication and interactions among different molecules, such as chemokines, growth factors, cytokines, and inflammatory factors [76,77]. The structure, activity, and evolution of cells in the TME tend to mirror the processes of inflammation and wound healing. One reason why a parallel between inflammation/wound healing and cancer is often drawn is the fact that these processes may involve the downstream activation of oncogenic mutations, immune cells, and vasculature structures [75,76,77]. The TME not only provides a physical scaffold that leads to the development of cancer, but also contains various growth factors, such as chemokines and angiogenic factors, that interact with various cell surface receptors.

The biophysics and role of activin/TGFβ have also been examined in cancer-related research. Cancer genome sequencing studies have revealed that components of the TGFβ superfamily are mutated to drive tumorigenesis. Additionally, evidence from recent studies has shown that cancer development can be affected by mutations in the TGFβII receptor (TGFBR2), SMAD4, and activin receptor 2A (ACVR2A) [77]. Both activin and TGFβ are critical components of the TME, and are usually involved in the successful regulation of cell differentiation, migration, proliferation, and apoptosis. In patients with colon cancer, the TGFβ superfamily tends to be growth suppressive, but in the advanced stages of the disease, cells expressing these particular proteins may be linked to a poor prognosis. Published research has also shown that TGFβ can induce activin secretion by tumor stromal cells and promote metastatic behavior in epithelial cells [74].

Initially, activin was considered to promote the release of follicle-stimulating hormone from the pituitary gland [66,73,74]. In addition, its roles in reproduction have been highlighted. More recently, activin has attracted the attention of cancer researchers, with a focus on how it affects inflammation, immunity, fibrosis, and angiogenesis. Evidence from laboratory studies revealed that activin is an important regulator of skin carcinogenesis and wound healing. Activin signaling is a critical aspect of metastatic pancreatic cancer progression [66]; pancreatic cancer patients have high serum levels of activin, a trend that leads to a poor prognosis. Animal model research has revealed that the overexpression of activin can lead to the formation of larger tumors and a significant reduction in weight, which is indicative of cancer cachexia [75]. Due to the considerable importance of activin in disease development, further investigations are needed to determine how it can be targeted for cancer treatment [75]. Moreover, the biochemical signaling processes that drive activin function provide a new area of exploration for new treatment options for cancer management.

### 4.11. Cancer Microbiome

The microbiomes that normally colonize the epithelial surfaces of the body are known to produce metabolites and molecules that have direct systematic and local effects on the onset of cancer [61]. Additionally, these molecules can influence disease progression and determine the patient response to therapy. Several studies have been performed using mouse models to explore the relationship between cancer phenotypes and microbial species [61,62]. Recent investigations show that microbiota have a profound effect on the efficacy of cancer treatments such as immunotherapy [61,62]. Moreover, researchers have attempted to identify the specific microbes that affect cancer phenotypes while also elucidating the underlying mechanism. The outcomes of these investigations have provided new strategies for developing microbiota-based therapies for different types of cancer.

The impact of gut microbiota on the development of cancer usually depends on interactions among the immune system, TME components, and microbiota [61]. A review of previous studies shows that cancer microbiota and other microorganisms in the TME influence processes such as biotransformation and xenobiotic metabolism that affect tumorigenesis [62]. These typically complex and multilayered processes determine the rate at which cancer cells grow and spread in the body. Thus, cancer microbiota are considered an essential factor that can influence the health of cancer patients and determine how they respond to treatment [62,63]. Several investigations are underway to explore the effect and role of microbiota in disease initiation and progression [77,78,79]. The success of fecal microbiota transplantation (FMT) in the management of *Clostridium difficile* infection will foster success in treating complex cancer cases [77,78,79].

### 4.12. Strategies for Cotreating the Tumor Microenvironment

There is overwhelming research evidence that the composition of the TME is heterogeneous [80]. However, some specific cells and mediators can be targeted in all types of cancer to improve the health and wellbeing of patients. For instance, researchers have shown that targeting CTLA4 with antibodies through immunotherapy approaches can help treat advanced cancer [80]. Moreover, angiogenesis inhibitors and multityrosine kinase inhibitors can affect VEGF signaling in various types of human cancer [80]. Ongoing clinical trials aim to target, eliminate, and reprogram myeloid cells in the TME to improve patient response to chemotherapy and ensure that the drugs reach the desired locations [81]. Researchers are also striving to understand cancer-associated inflammation as a way of identifying targeted agents, such as therapeutic antibodies, with potential efficacy in managing cancer. Antibodies that bind to PD-1 without cell-activation can prevent immune cells from inactivation through PD-1Ligand (immune checkpoint therapy).

Research evidence has revealed that nonmalignant cancer cells in the TME account for approximately 50 percent of tumor masses and associated metastases [80]. However, the actual functions of specific components are not well understood. Furthermore, there is limited information on how the TME evolves during the course of cancer development, progression, and treatment [81,82,83]. Nonetheless, heterogeneous mutations in cells at various tumor sites and in their constituents can potentially be targeted to treat cancer. The significance of the TME in the development of new cancer management regimens is becoming apparent, with increased research being done to identify its composition [84,85]. Targeting specific components in tumor sites may help eliminate the protumorigenic components and manage the immunosuppressive components linked to tumor growth [85]. Additionally, there is a need to determine how new antigens that reawaken the immune system can be used to manage cancer. For a summary of the most important potential therapies regarding the TME please see Table 3.

## 5. Conclusions

The TME is a complex ecology consisting of cells that evolve with cancer cells and provide support during malignant transformation. In addition to malignant cells, the TME contains cells of the lymphatics, tumor vasculature, and immune system, as well as adipocytes and fibroblasts. These cells can be recruited to the tumor, and are present at all phases of tumor development and progression. This systematic review has revealed that the cells in the TME possess immunologic phenotypes and capabilities that influence disease progression. Therefore, further studies should assess how targeting cells in the TME, such as CAFs, B lymphocytes, and Tregs, can contribute to the management of cancer by altering or stopping tumor progression.

The TME has multiple effects on tumor initiation, development, and progression. It contains cells and molecules that can increase the stemness of tumor cells, promote angiogenesis, mediate migration, induce drug resistance, and suppress the immune system. An in-depth understanding of the TME and its roles and associated molecules will offer crucial insights into the biological behavior of different types of tumors. Additionally, such data will provide an essential foundation for developing TME-based therapeutics to manage and control carcinomas. Protumorigenic processes and molecules in the TME are crucial targets for new cancer therapies.

## Figures and Tables

**Table 1:** Studies selected at the end of the search process.

Study	Purpose	Design	Summary of Findings
Grivennikov, Green, and Karin 2010 [8]	To examine the mechanisms governing the effects of immunity and inflammation on tumor development	Systematic review	Inflammatory responses play a critical role in various stages of tumor development, including promotion, initiation, metastasis, and invasion.
Spill et al. (2016) [9]	To explore the importance and influence of the tumor microenvironment in cancer development and progression	Systematic review	The tumor microenvironment plays a critical role in cancer development and progression. In particular, biochemical cues within the tumor microenvironment can affect cell behavior, metastatic potential, and cancer stem cell characteristics.
Del Prete et al. (2017) [10]	To examine the role of tumor-associated immune cells in tumor initiation, growth, and dissemination	Systematic review	The tumor microenvironment contains both malignant and nonmalignant cells and other soluble mediators that influence tumor growth.
LeBleu [11]	To provide an overview of the different cell types and physiological parameters of the tumor microenvironment and how they affect tumor development	Systematic review	The tumor microenvironment represents a complex and dynamic milieu of both cellular and acellular components with synergistic activity and function in cancer progression.
Korneev et al. (2017) [12]	To examine the role of Toll-like receptor (TLR) 4 signaling in cancer development and the creation of a protumorigenic microenvironment	Systematic review	Tumors interact closely and continuously with the surrounding microenvironment and organs via the lymphatic or circulatory system. Thus, tumor cells can influence the microenvironment through the release of extracellular signals, such as paracrine signals, to induce peripheral immune tolerance and support tumor angiogenesis.
Feig et al. (2013) [13]	To study FAP-expressing carcinoma-associated fibroblasts	Animal model study	The re-expression of Kras in the pancreas can influence the growth and development of both metastatic and invasive carcinoma.
Rupp et al. (2014) [14]	To study IGFBP7 as unique tumor stroma marker with tumor growth-promoting effects	Experimental design	IGFBP7 affects physiological homeostasis by controlling tumorigenesis, tumor cell growth, metastasis, and invasion. The results showed that the stroma contributes to the anchorage-independent growth and progression of mesenchymal cells.
Chen et al. (2015) [15]	To examine new research evidence on the biology of the tumor microenvironment	Systematic review	The tumor microenvironment can affect cancer growth and development. Thus, targeting the tumor microenvironment can help enhance acquired resistance, improve therapeutic efficacy, and prevent metastasis.
Pujari and Vidya (2015) [16]	To analyze the biology of the tumor microenvironment	Systematic review	The presence of immune cells in the tumor microenvironment and the process of angiogenesis lead to the generation of cancer stem cells while providing a complex signaling environment.
Pylayeva-Gupta et al. (2012) [17]	To study the interactions between PanINs and tumor microenvironment	Systematic review	Suppressing the production of GM-CSF can hinder the growth of PDECs and the expression of Kras.
Bussard et al. (2016) [18]	To examine current research evidence on the origin and effect of recruited host stroma on tumor progression and development	Systematic review	The tumor microenvironment is a mixture of tumor cells and endogenous host stroma that influences cancer growth and development.
Pottier et al. (2015) [19]	To study the significance of the tumor microenvironment in the treatment and management of cancer	Systematic review	The tumor microenvironment can influence disease prognosis and antitumor immunity. Furthermore, it can influence the outcomes and effectiveness of cancer management therapies.
Watnick RS (2012) [20]	To determine the role of the tumor microenvironment with regard to the regulation of angiogenesis	Systematic review	The tumor-associated stroma plays a key role in cancer formation and development. Furthermore, intracellular signaling in the tumor microenvironment can regulate angiogenesis.
Angell and Galon (2013) [21]	To examine the role of predictive and immune markers of cancer development	Systematic review	The complex interactions between predictive and immune markers and other cells in the tumor microenvironment can influence cancer development, progression, and patient prognosis.
Lv et al. (2012) [22]	To study the accumulation and prognostic value of tumor-infiltrating cells that produce IL-17	Experimental design	Tumor-infiltrating cells that produce IL-17 play a protective role in tumor development.
Tanaka and Iwakiri (2012) [23]	To study the structure and function of the vascular and hepatic lymphatic systems	Systematic review	The study reported that the vascular and lymphatic systems play essential physiological roles in the human body that can influence tumor development.
Schioppa et al. (2011) [24]	To examine regulatory B cells and the protumorigenic effects of TNF-α	Experimental design	The study showed that TNF-α can mediate protumorigenic actions through regulatory B cells.
Vivier et al. (2012) [25]	To explore and study biomarkers of metastatic colorectal cancer	Systematic review	NK cells are critical biomarkers that play a key role in fighting the growth and development of colorectal cancer.
Meredith et al. (2012) [26]	To examine the expression of the zinc finger transcription factor zDC (Zbtb46, Btbd4)	Systematic review	Dendritic cells in the tumor microenvironment contribute to antigen presentation and processing by acting as messengers between the adaptive and innate systems. Furthermore, the results showed that CD11c-expressing non-cDCs can help initiate immunity to tumors and pathogens.
Yoon et al. (2010) [27]	To explore the ability of GATA3 expression to predict breast cancer survival	Systematic review	Higher levels of GATA3 predict better survival in women with breast cancer. In contrast, lower levels of GATA3 predict disease-related death.
Tietze J et al. (2012) [28]	To characterize CD8+ T cells and examine the role of antigen-specificity in effector function	Animal model	The cytokine-mediated stimulation of CD8+ T cells leads to antigen-nonspecific expansion.
Mao et al. (2013) [29]	To examine current knowledge on the tumor stroma in breast cancer patients	Systematic review	The tumor microenvironment can influence the growth and development of breast cancer. The tumor stroma contains critical components such as leukocytes, TAMs, and CAFs that influence malignant processes.
Hofer and Tuan (2016) [30]	To examine the clinical efficacy of MSCs	Systematic review	The activities of MSCs are intricately controlled and can influence tissue regeneration.
Plaks, Kong, and Werb (2015) [31]	To explore tumor initiation, tumor progression, and cancer therapy	Systematic review	Cancer cells originate from cells that have gained tumor-initiating capacity. The tumor microenvironment affects the growth of these cells and their impact on surrounding tissue.
Papaccio et al. (2017) [32]	To study the role of cancer stem cells in cancer development and progression	Systematic review	Cancer stem cells play a key role in the initiation and progression of tumors. They have self-renewal and differentiating capacities.
Fozza and Longinotti (2011) [33]	To examine the process that modulates T cell recruitment to the tumor and lymph node microenvironments	Systematic review	The tumor microenvironment is composed of nonneoplastic cells with unique functions and peculiar phenotypic features. These cells modulate T cell recruitment to the tumor microenvironment.
Andreu et al. (2010) [34]	To examine how FcRγ activation influences and regulates inflammation-associated squamous carcinogenesis	Animal model study	The study shows that B cells, activating FcγRs, and humoral immunity facilitate the creation of chronic inflammatory programs that can promote and influence de novo carcinogenesis.
Martinet et al. (2015) [35]	To study how DNAM-1 expression affects NK cell maturation.	Experimental design	NK cells play a key role in cancer surveillance and pathogen defense. The activation and expression of the receptor DNAM-1 results in two NK cell subsets (DNAM-1(+) and DNAM-1(−)). Thus, DNAM-1 expression indicates an alternative program of NK cell maturation.
Das et al. (2013) [36]	To study the role of the adaptor molecule SAP in lytic synapse formation	Animal model study	Using an animal model, researchers showed that SAP plays a key role in lytic synapse formation and invariant NKT cell cytotoxicity.

FAP—fibroblast-activation protein; GM-CSF—granulocyte macrophage colony stimulating factor; MSCs—mesenchymal stem cell; PanINs—pancreatic precancerous lesions; PDECs—primary ductal epithelial cells.

**Table 2:** Cells in the tumor microenvironment.

Cell Players	Main Markers or Types	Primary Functions
T lymphocytes	CD8^+^ and CD4^+^	Some are protumorigenic, while others are tumor restrictive [43].
B lymphocytes	Regulatory B cells and B10 cells	They contribute to the regulation of tumor cell survival and proliferation and the development of treatment resistance. In addition, these cells have been linked to the process of fostering immune escape [4,23].
NK and NKT cells	NKG2 receptors, Ly49 receptors, NK1, CD94, C57BL/6, CD161, NKG2D, CD56, and NKG2A	NK and NKT cells use inhibitory, adhesion, activating, and cytokine receptors to identify cellular targets and healthy spare cells [48].
Macrophages	M1 and M2 macrophages	They create a stroma that is supportive of neoplastic cell invasion and expansion [6,7].
Macrophages M1	antitumorigenic	
Macrophages M2	immunosuppressive and pro-tumorigenic	As M2 macrophages are immune-suppressive, they can promote tumor progression
Cancer-associated fibroblasts	α-Smooth muscle actin, fibroblast activation protein, vimentin, desmin, and PDGFR α and β	They contribute to tumor cell proliferation by maintaining continuous propagation and growth signals at primary and metastatic sites [53,54].
Cancer stem cells	Tumor stem cells and DPSCs	They support tumorigenesis through unique homing abilities to primary and metastatic sites [63].
Chemokines	CXCL14 and CXCL12	They are usually overexpressed on myofibroblasts and myoepithelial cells. These molecules can bind epithelial cell receptors to increase cell migration, invasion, and proliferation [65,66,67].
Integrins	αMβ2, αXβ2, αLβ2, αDβ2, α4β7, and αEβ7	They bind to the extracellular matrix in the TME [14].
Selectins	Epidermal growth factor (EGF)-like motif, ST3Gal6, P-selectin	These are vital vascular adhesion molecules that affect the development of cells [2,12].
Cadherins	Protocadherin, desmogleins, and desmocollins	These molecules mediate the formation of homophilic bonds in a calcium-dependent manner [7].
Tregs	CD4, FOXP3, and CD25	These cells promote the generation and function of vaccine-elicited CD8+ memory T cells [34,69].
Immunoglobulin superfamily (IgSF)	Cell surface antigen receptors, coreceptors, and costimulatory molecules	These molecules mediate the formation of both heterophilic and homophilic bonds [46,51].
Bone marrow derived cells (BMDC)	BMDCs have several tumor growth-promoting functions.	Tumor growth promoting functions include expression of growth factors, promotion of tumor vessel formation and creation of tumor stem cell niches
Myeloid derived suppressor cells (MDSC)	MDSCs expand in pathological situations such as cancer, as a result of an altered hematopoiesis	MDSCs possess strong immunosuppressive activity especially on myeloid cells.

**Table 3:** Summary of potential therapies.

Treatment Option	Details
Targeting tumor acidosis	Tumor acidosis controls malignant behavior, determines the rates of metastasis and invasion, and regulates metabolic adaptations in cancer [75,76,77].
Targeting LAMP2	LAMP2 is upregulated by acidic proteolysis during cancer development [75,76].
Immune checkpoint inhibition	PD-L1 binding to PD-1 can prevent T cells from killing tumor cells. Further studies have revealed that inhibitors of the association between PD-L1 and PD-1 can decrease cancer cell evasion from immune attacks [13].
Targeting ECM-integrin signaling	Targeting ECM-integrin signaling entails managing the activity of integrins as primary cell surface receptors for different ECM proteins [78,79]. Integrins affect cancer cell differentiation, proliferation, and survival [79].
TGF-β and activin	TGFβII receptor (TGFBR2), SMAD4, and activin receptor 2A (ACVR2A) receptor mutations affect the development of cancer [74,77]. The TGFβ superfamily is growth-suppressive and can slow cancer progression [74,77].
Cancer microbiome	Cancer microbiota affect the rate of disease progression [61,62].
Fecal microbiota transplantation (FMT)	FMT has been identified as a novel intervention for managing complex cancer cases [77,78,79].
Targeting CTLA4 antibodies	Immunotherapy approaches can help treat cases of advanced cancer [80].
Angiogenesis and multityrosine kinase inhibitors	These therapies can affect VEGF signaling in a variety of human cancer types [80].
